# Parameter selection and optimization of a computational network model of blood flow in single-ventricle patients

**DOI:** 10.1098/rsif.2024.0663

**Published:** 2025-02-27

**Authors:** Alyssa M. Taylor-LaPole, L. Mihaela Paun, Dan Lior, Justin D. Weigand, Charles Puelz, Mette S. Olufsen

**Affiliations:** ^1^ Department of Mathematics, North Carolina State University, Raleigh, NC, USA; ^2^ School of Mathematics and Statistics, University of Glasgow, Glasgow, UK; ^3^ Division of Cardiology, Department of Pediatrics, Baylor College of Medicine and Texas Children’s Hospital, Houston, TX, USA; ^4^ Department of Mathematics, University of Houston, Houston, TX, USA

**Keywords:** parameter inference, patient-specific modelling, cardiovascular fluid dynamics, medical image analysis, hypoplastic left heart syndrome, double outlet right ventricle

## Abstract

Hypoplastic left heart syndrome (HLHS) is a congenital heart disease responsible for 23% of infant cardiac deaths each year in the United States. HLHS patients are born with an underdeveloped left heart, requiring several surgeries to reconstruct the aorta and create a single-ventricle circuit known as the Fontan circulation. While survival into early adulthood is becoming more common, Fontan patients often have a reduced cardiac output, putting them at risk for a multitude of complications. These patients are monitored using chest and neck magnetic resonance imaging (MRI), but their scans do not capture energy loss, pressure, wave intensity or haemodynamics beyond the imaged region. This study develops a framework for predicting these missing features by combining imaging data and computational fluid dynamics (CFD) models. Predicted features from models of HLHS patients are compared with those from control patients with a double outlet right ventricle (DORV). We infer patient-specific parameters through the proposed framework. In the calibrated model, we predict pressure, flow, wave intensity (WI) and wall shear stress (WSS). Results reveal that HLHS patients have lower compliance than DORV patients, resulting in lower WSS and higher WI in the ascending aorta and increased WSS and decreased WI in the descending aorta.

## 1. Introduction

**Figure 1.**
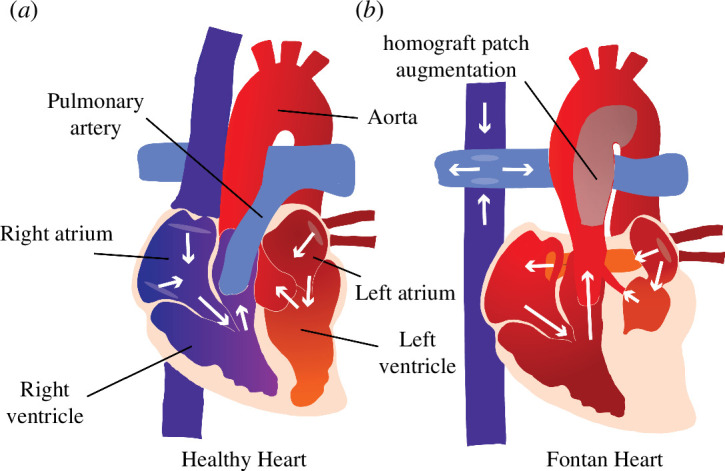
Comparison of healthy (*a*) and single-ventricle (*b*) hearts and vessels. The homographic patch augmentation generated on HLHS patients’ ascending aorta is highlighted in grey in panel (*b*). DORV patients have a Fontan circulation but do not need aortic reconstruction. The white arrows indicate the direction of blood flow.

Hypoplastic left heart syndrome (HLHS) is a congenital heart disease responsible for 23 % of infant cardiac deaths and up to 9 % of congenital heart disease cases each year in the United States [1]. The disease arises in infants with an underdeveloped left heart, preventing adequate transport of oxygenated blood to the systemic circulation [2]. Characteristics of HLHS include an underdeveloped left ventricle and ascending aorta, as well as small or missing aortic and mitral valves. Three surgeries are performed over the patient’s first 2–3 years of life, resulting in a Fontan circuit (figure 1), a fully functioning, univentricular circulation. The first surgical stage involves moving the aorta from the left to the right ventricle and widening it with tissue from the pulmonary artery. This surgically modified aorta is hereafter referred to as the ‘reconstructed’ aorta. Next, the venae cavae are removed from the right atrium and attached to the pulmonary artery. As a result, flow to the pulmonary circulation is achieved by passive transport through the systemic veins [3–5]. The Fontan circuit has near-normal arterial oxygen saturation. However, the lack of a pump pushing blood into the pulmonary vasculature causes a bottleneck effect, increasing pulmonary impedance and decreasing venous return to the heart [6]. The single-pump system degenerates over time due to vascular remodelling accentuated by the system’s attempt to compensate for reduced cardiac output [5–7]. Complications of the Fontan physiology include reduced cerebral and gut perfusion, increasing the risk of stroke [8] and the development of Fontan-associated liver disease (FALD) [9,10]. The study by Saiki *et al*. [8] shows that increased stiffness of the aorta, head and neck vessels in HLHS patients with reconstructed aortas reduces blood flow to the brain. This reduced flow, combined with increased arterial stiffness, is associated with ischaemic stroke [11]. Regarding FALD, the single pump system decreases both supply and drainage of blood in the liver. Hypertension within the liver vasculature typically occurs 5–10 years after Fontan surgery [9]. Reduced cardiac output and passive venous flow lead to liver fibrosis, a characteristic of FALD. With a heart transplant, FALD is reversible if caught early. Advanced FALD is irreversible and can lead to organ failure [9].

Another single-ventricle pathology is a double outlet right ventricle (DORV), also treated by establishing a Fontan circulation. DORV patients have a non-functioning left ventricle but are born with a fully functioning aorta attached to the right ventricle. This physiology makes aortic reconstruction unnecessary [12]. Figure 1 shows a healthy (*a*) and a single-ventricle (*b*) heart and circulation. The grey patch on the aorta indicates the reconstructed portion, required in HLHS but not DORV patients. Studies [13,14] suggest that DORV patients undergo less vascular remodelling and, therefore, have better cardiac function. Rutka *et al*. [13] finds that Fontan patients with reconstructed aortas had poorer long-term survival and Sano *et al*. [14] notes that these patients have an increased need for surgical intervention and increased mortality rates.

Single-ventricle patients are monitored throughout life to assess the function of their ventricular pump [1]. Typically, this is done via time-resolved magnetic resonance imaging (4D-MRI) [15–17] and magnetic resonance angiography (MRA) of vessels in the neck and chest. 4D-MRI provides time-resolved blood velocity fields in large vessels, while MRA provides a high-fidelity image of the vessel anatomy. These imaging sequences do not measure energy loss, blood pressure, wave intensity or haemodynamics outside of the imaged region. Additional haemodynamic information can be predicted by combining imaging data with computational fluid dynamics (CFD) modelling [18–22].

Most computational work on the Fontan circulation focuses on venous blood flow. This work includes studies on the haemodynamics and function of the total cavopulmonary connection [23–25]. However, computational studies assessing the Fontan circuit from the systemic arterial perspective are scarce. A few have examined systemic arterial haemodynamics in individual patients. This includes the study by Taylor-LaPole *et al*. [18] using a one-dimensional CFD model to compare DORV and HLHS flow and pressure wave propagation in the cerebral and gut vasculature under rest and exercise conditions. Their study is promising, but results are calibrated manually and only include a single DORV and HLHS patient pair. The study by Puelz *et al*. [26] uses a one-dimensional CFD model to explore the effects of two different Fontan modifications (fenestration versus hepatic vein exclusion) on blood flow to the liver and intestines. Their study compares model predictions with ranges of clinical data obtained from the literature. Still, they lack a systematic methodology to determine model parameters. These studies successfully build patient-specific networks and predict haemodynamics outside the imaged region, demonstrating the importance of calibrating models to data. However, they lack a systematic methodology to determine model parameters.

This limitation is addressed by combining imaging and haemodynamic data with a one-dimensional CFD model. The model is calibrated to patient-specific data using sensitivity analysis and parameter inference, an improvement over previous studies that rely on manual tuning of parameters. The approach presented here is used to predict haemodynamics in four matched HLHS and DORV patient pairs.

Calibration of the model is crucial, especially when it is used for predictions in a clinical setting. For this purpose, an identifiable subset of parameters is needed. Calibrating the model by estimating identifiable parameters provides unique patient-specific biomarkers which can be compared between patients. Two steps are used to determine an identifiable parameter subset: sensitivity analyses (local and global), which determine the effect of a given parameter on a specified quantity of interest [27], and subset selection, which characterizes interactions among parameters [28,29]. Several recent studies use these methodologies. Schiavazzi *et al*. [30] combine local and global sensitivity analyses to find a set of parameters that fit a zero-dimensional model to data from single-ventricle patients. This study demonstrates that using both techniques leads to consistent and accurate subset selection. The study by Colebank *et al*. [27] uses local and global sensitivity analyses to determine the most influential parameters. They combine results with covariance analysis to quantify parameter interactions and further reduce the parameter subset to be inferred. The model is calibrated using experimental data, but pressure data is available only at one location. Colunga *et al*. [31] use local and global sensitivity analyses for subset selection. Their study incorporated multistart inference to ensure identifiability and low coefficient of variation for all parameters.

Inspired by these studies, we use local and global sensitivity analyses to determine parameter influence and covariance analysis to identify correlated parameters. We then use multistart inference to estimate the identifiable parameters. Once calibrated, we predict haemodynamic quantities, including pressure, flow, wave intensity and wall shear stress to compare cardiac function between HLHS and DORV patients. This study aims to create an efficient and accurate parameter inference pipeline for single-ventricle patients. Parameter inference and model calibration are crucial to ensure accurate haemodynamic insights that clinicians can investigate for each patient. To our knowledge, this is the first study that uses multiple data sets to calibrate a one-dimensional, patient-specific CFD model for single-ventricle patients.

## 2. Methods

**Figure 2.**
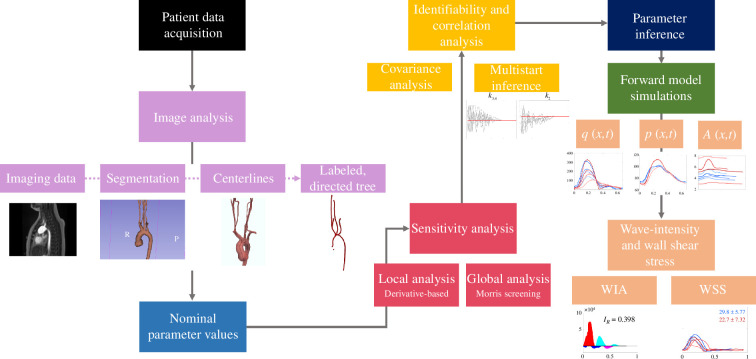
Workflow of the methods implemented in this study. We begin with patient data acquisition, consisting of biometrics, 4D-MRIs and MRAs. We then perform image analysis to obtain patient geometries. We set nominal parameter values for each patient and perform a sensitivity analysis. Once we have determined influential parameters, we use covariance analysis to choose a subset of identifiable parameters estimated using multistart parameter inference. Estimated parameters are used for forward model simulations for each patient.

Medical imaging and haemodynamic data are combined to construct a patient-specific one-dimensional arterial network model. MRA [15] are segmented, creating a three-dimensional representation of vessels within the imaged region. For each HLHS and DORV patient, we extract vessel dimensions (radius and length) and vessel connectivity. The MRA images are registered to the 4D-MRI images, allowing for the extraction of flow waveforms in the aorta, head and neck vessels using the high-fidelity MRA anatomy. The network includes vessels in the imaged region extended by one generation. This construction ensures sufficient resolution of wave reflections. We solve a one-dimensional CFD model in this network and use sensitivity analyses and subset selection to determine a subset of identifiable parameters. Parameter inference uses a gradient-based optimization scheme to minimize the residual sum of squares (RSS). A full workflow of our methods can be seen in figure 2.

### 2.1. Data acquisition

Data include four pairs of age- and sized-matched HLHS and DORV patients seen at Texas Children’s Hospital, Houston, TX. Data collection was approved by the Baylor College of Medicine Institutional Review Board (H-46224: ‘Four-Dimensional Flow Cardiovascular Magnetic Resonance for the Assessment of Aortic Arch Properties in Single Ventricle Patients’). To isolate differences in flow properties based on native versus reconstructed aortas, single-ventricle morphologies were selected to have the same dominant single right ventricle. The DORV group includes patients with single-ventricle anatomy, right ventricular morphology and native arches. The HLHS group includes patients with single-ventricle anatomy, right ventricular morphology and reconstructed aortas. Each cohort has similar atrioventricular valve regurgitation, achieved total cavopulmonary anastomosis (non-fenestrated), and have preserved right ventricular systolic function. Exclusion criteria include patients with aortic surgery beyond the initial Norwood, significant collateral burden of less than 40%, systemic hypertension and those with heart failure. Patients were matched across cohorts (DORV to HLHS) based on body surface area and age.

#### 2.1.1. Imaging and pressure data

Cuff pressures are measured in the supine position. Images are acquired on a 1.5 T Siemens Aera magnet. MRA images are acquired in the sagittal plane and contain a reconstructed voxel size of 1.2 mm^3^. 4D-MRI images are acquired during free breathing with a slice thickness of 1–2.5 mm and encoding velocity of 10% larger than the highest anticipated velocity in the aorta. The imaged region contains the ascending aorta, aortic arch, descending thoracic aorta and innominate artery vessels (Figure 5).

Patient characteristics, including age (years), height (cm), sex (m/f), weight (kg), systolic and diastolic blood pressure (mmHg, measured with a sphygmomanometer) and cardiac output (l min^−1^), are listed in table 1.

**Table 1.** Patient data including age, height, weight, gender, systolic and diastolic pressures, cardiac output (CO) and cardiac cycle length ( T ).

pair	patient	age	height (cm)	weight (kg)	gender	pressure (mmHg)	CO (l min^−1^)	T (s)
pair 1	DORV 1	16	171.7	52.6	M	105/57	4.0	0.802
	HLHS 1	18	159.0	57.5	M	129/72	5.4	0.942
pair 2	DORV 2	12	154.3	59.6	F	110/67	4.0	0.658
	HLHS 2	11	151.4	62.0	M	116/65	3.9	0.615
pair 3	DORV 3	11	136.4	32.7	M	119/60	3.3	0.774
	HLHS 3	13	148.0	38.1	M	99/53	2.8	0.933
pair 4	DORV 4	12	148.5	42.3	F	112/6	3.7	0.598
	HLHS 4	14	163.0	53.7	M	116/60	3.2	0.605

#### 2.1.2. Centrelines

**Figure 3.**
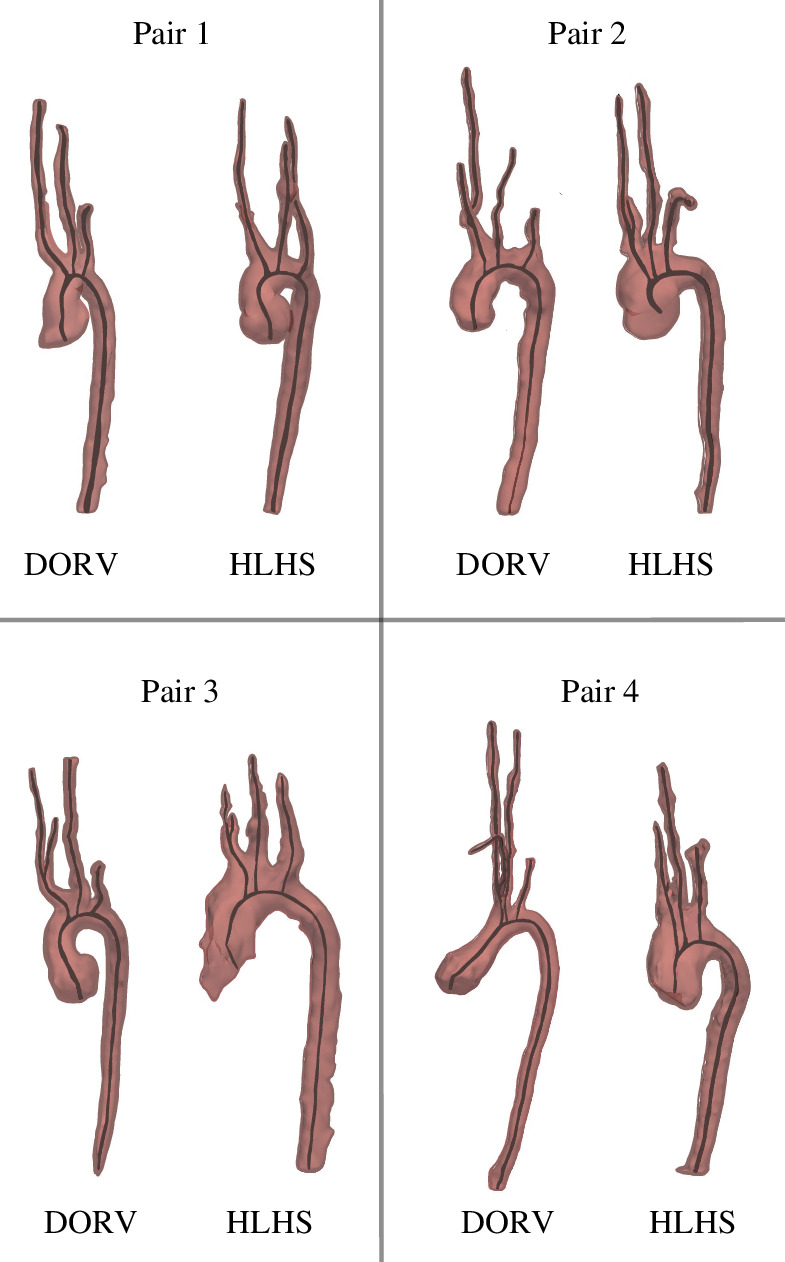
Segmentations and centrelines for each patient are shown in their respective pairs.

Using the Vascular Modelling Toolkit (VMTK), centrelines are generated from maximally inscribed spheres (figure 3). At each point along the centreline, the vessel radius is obtained from the associated sphere [32]. Users manually select inlet and outlet points. VMTK recursively generates centrelines, beginning at the outlets and determining pathways to the inlet. Junctions are defined as the points where two centrelines intersect. This procedure does not place the junction point at the barycentre for most vessels, especially those branching from the aorta. To correct this, we use an in-house algorithm [33] detailed in the electronic supplementary material.

#### 2.1.3 Image registration

**Figure 4.**
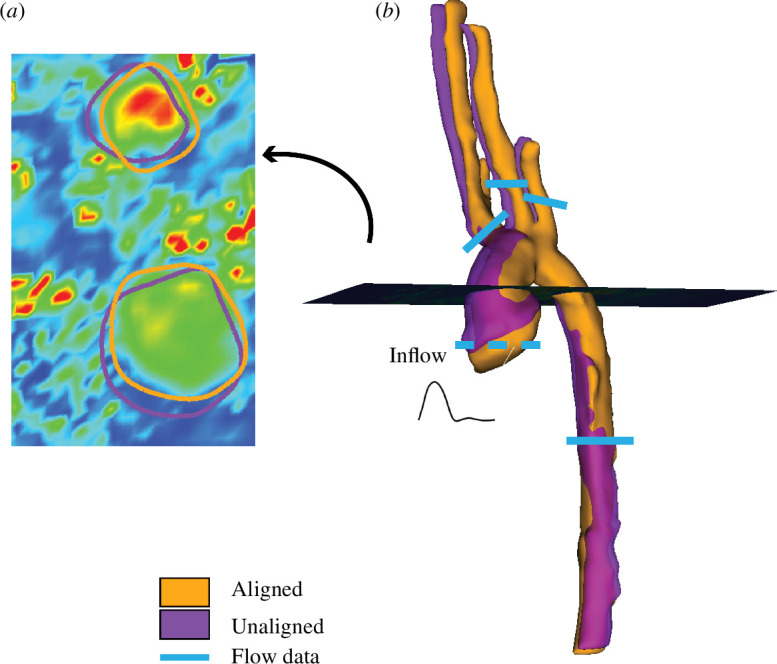
Before and after registering the MRA to the 4D-MRI. Panel (*a*) shows velocities from the 4D-MRI in the unaligned (purple circles) versus the aligned (orange circles) network. Panel (*b*) shows aligned (orange) MRA segmentation transposed on the unaligned segmentation (pink). The velocities in (*a*) correspond to the slice through the segmentations in (*b*). Blue lines denote where flow data were obtained from each patient. The dashed blue line shows where the inflow profile was obtained.

Volumetric flow waveforms are extracted from the 4D-MRI images. The 4D-MRI sequence measures the time-resolved blood velocity field at each voxel in the imaged region with a sampling frequency of 19–28 times throughout a cardiac cycle. 4D-MRIs provide vascular geometry, but only at certain phases of the cardiac cycle and with low spatial resolution. Hence, both an MRA for the anatomy and a 4D-MRI for the velocity field are acquired for each patient. These two images are obtained in the same session, but shifting and deformation may occur between scans caused by patient movement or misalignment within the respiratory cycles. Without correction, this can lead to inaccurate blood flow and volume estimates. To compensate, the MRA and 4D-MRI are aligned via an image registration procedure (shown in figure 4) [34]. A detailed description is given in the electronic supplementary material.

#### 2.1.4. Volumetric flow data

Flow waveforms are extracted from the 4D-MRI image using the aligned MRA segmentation. See the electronic supplementary material for a detailed description.

The 4D-MRI velocity field is generally not divergence-free, in part because of noise in the measurements and averaging performed over several cardiac cycles. Thus, the sum of the flows in the branching vessels does not equal the flow in the ascending aorta. The one-dimensional CFD model assumes that blood is incompressible, implying that flow through vessel junctions is conserved. To ensure convergence of the optimization, we impose volume conservation by scaling the flow waveforms extracted from the 4D-MRI images. The scaling uses a linear system to calculate the smallest possible scale factors for the flow values that are needed to enforce mass conservation. The cardiac output of the ascending aorta is held fixed, and flows in the peripheral branching vessels are scaled by


(2.1) $$q_{\text{inlet}}=\sum_{i=1}^{4}\left(1+\alpha_{i}\right)q_{i},$$

where $q_{\text{inlet}}$ denotes the flow through the ascending aorta, $\alpha_{i}$ is the scaling factor for the ith vessel and $q_{i}$ is flow in the ith vessel. A linear system for the scaling factors $\alpha_{i}$ is constructed from the constrained optimization problem, minimizing


(2.2) $$\begin{array}{lcr} & f=\lambda \left(\sum_{i=1}^{n}\left(\left(1+\alpha_{i}\right)q_{i}\right)-q_{\text{inlet}}\right)+\frac{1}{2n}\sum_{i=1}^{n}\alpha_{i}^{2}, & \end{array}$$

where n=4 . See the electronic supplementary material for a complete description. It should be noted that the flow waveforms are determined by extracting five flow waveforms from the 4D-MRI image that lie close to the centreline and are within a straight section of the vessel. These waveforms are averaged to create a representative flow waveform.

### 2.2. Network generation

**Figure 5.**
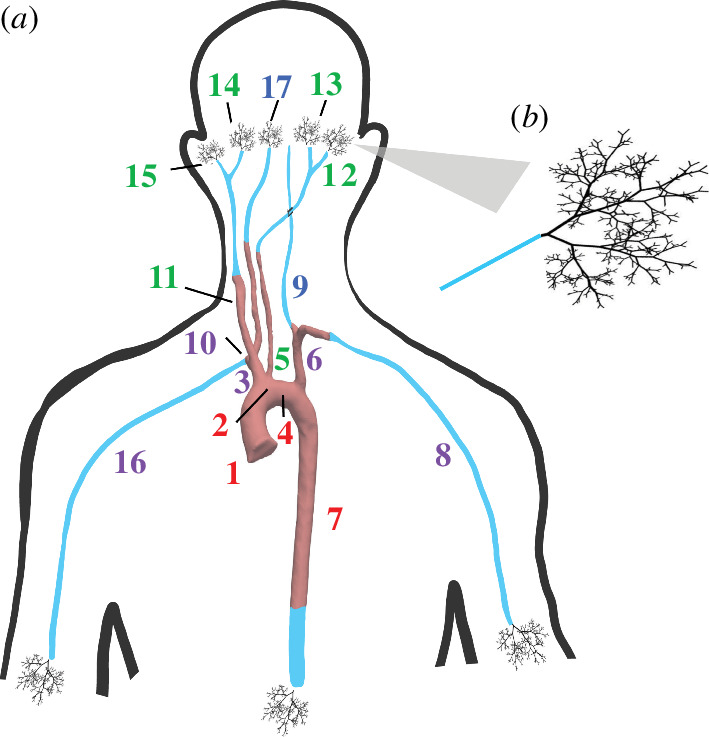
Network used in the fluids model. (*a*) Arteries extracted from the MRA images (dark red) and extended vessels outside of the imaged region (cyan). Small vessel networks (black), represented by structured trees are attached to the end of each large terminal vessel. (*b*) Example structured tree including vessels down to the arteriolar level. Vessel numbers correspond to those given in table 2. The vessel number colours indicate the parameter group used for parameter inference described in §2.4. Red vessel numbers represent group 1, purple group 2, green group 3 and blue group 4.

A labelled directed graph is generated for each patient using in-house algorithms [35,36], extracting vessel radii and lengths from the VMTK output. The centrelines are defined as edges, containing x,y,z coordinates along the centreline and junctions as nodes. Vessel radii are specified at each point along an edge from the radius of the maximally inscribed sphere. The vessel length is calculated as the sum of the Euclidean distances between the x,y and z coordinates. Nodes shared between edges spanning more than one vessel are called ‘junction nodes’. Terminal vessels are defined as those with no further branching. A connectivity matrix is used to specify how the vessels are connected in the network. For example, in figure 5, vessel 1 (the ascending aorta) forms a junction with vessel 2 (the aortic arch) and vessel 3 (the innominate artery).

**Table 2.** Vessel dimensions. Values denote the average of each measurement ± one s.d. Vessel numbers correspond to those shown in figure 5. Exact dimensions for individual patients can be found in the electronic supplementary material.

		DORV			HLHS		
number	name	L(cm)	$R_{\text{in}}\;(cm)$	$R_{\text{out}}\;(cm)$	L(cm)	$R_{\text{in}}\;(cm)$	$R_{\text{out}}\;(cm)$
1	Asc aorta	3.8±0.8	1.18±0.08	1.04±0.03	2.9±0.9	1.36±0.25	1.52±0.22
2	AA I	1.6±0.6	1.04±0.03	1.00±0.04	1.5±1.0	1.52±0.22	1.31±0.28
3	Innom	4.3±1.2	0.53±0.21	0.53±0.21	3.1±1.1	0.54±0.12	0.54±0.12
4	AA II	2.5±0.9	1.00±0.04	0.87±0.14	1.5±0.5	1.31±0.28	0.74±0.18
5	LCC	20.1±0.7	0.32±0.03	0.31±0.04	20.1±0.2	0.3±0.05	0.27±0.03
6,10	Subcl	3.3±0.1	0.36±0.09	0.34±0.11	3.5±0.4	0.21±0.04	0.28±0.02
7	Desc aorta	35.1±0.5	0.87±0.14	0.59±0.02	35.1±0.2	0.74±0.18	0.55±0.02
8	L Brachial	20.1±0.7	0.31±0.05	0.26±0.05	20.1±0.2	0.30±0.05	0.28±0.02
9,17	Vertebral	14.3±0.5	0.24±0.01	0.23±0.03	14.3±0.2	0.24±0.03	0.22±0.03
11	RCC	17.1±0.6	0.32±0.07	0.30±0.07	17.1±0.2	0.34±0.08	0.31±0.06
12,15	Ext C	17.1±0.6	0.27±0.08	0.24±0.11	17.1±0.2	0.28±0.06	0.26±0.07
13,14	Int C	17.0 ± 0.6	0.21±0.05	0.17±0.05	17.0±0.2	0.24±0.05	0.23±0.06
16	R Brachial	20.1±0.7	0.21±0.03	0.21±0.03	20.1±0.2	0.24±0.03	0.23±0.04

The generated network is a tree. The tree has a natural direction since flow moves from the heart towards the peripheral vasculature. The network is represented by a labelled directed graph (a tree), where labels include the vessel radii and lengths. The directed graph generated from the images is adjusted to move junctions to the barycentre and to determine representative vessel radii. This adjustment is done using an in-house recursive algorithm [33].

The network is extended beyond the imaged region to include one additional bifurcation to capture wave reflections in the model predictions [18]. In total, 17 vessels are included, with nine within the imaged region. Allometric scaling [37] is performed on vessel dimensions outside the imaged region. We scale values obtained from the literature [38] as


(2.3) $$\begin{array}{lcr} & L_{2}=L_{1}{\left(\frac{W_{1}}{W_{2}}\right)}^{\alpha }, & \end{array}$$

where α=0.35 , $W_{1}$ (kg) and $L_{1}$ (cm) are literature bodyweight and vessel length values, $W_{2}$ (kg) is the bodyweight of the patient and $L_{2}$ (cm) is the unknown vessel length. Vessel radii not specified in the imaged region are calculated similarly.

At the terminal ends of the large vessels, asymmetric structured trees are used as outlet boundary conditions for the fluids model. The structured trees represent arterial branching down to the arteriolar level (figure 5).

### 2.3. Fluid dynamics

A one-dimensional fluid dynamics model derived from the Navier–Stokes equations is used to compute pressure p(x,t) (mmHg), flow q(x,t) (ml s^−1^) and cross-sectional area A(x,t) (cm^2^). In the large vessels, the equations are solved explicitly, and in the small vessels, a wave equation is solved in the frequency domain to predict impedance at the root of the small vessels.

#### 2.3.1. Large vessels

We assume the blood to be incompressible, Newtonian and homogeneous with axially symmetric flow and the vessels to be long and thin. Under these assumptions, flow, pressure and vessel cross-sectional area satisfy mass conservation and momentum balance equations of the form


(2.4) $$\frac{\partial A}{\partial t}+\frac{\partial q}{\partial x}=0,$$

(2.5) $$\frac{\partial q}{\partial t}+\frac{\partial }{\partial x}\left(\frac{q^{2}}{A}\right)+\frac{A}{\rho }\frac{\partial p}{\partial x}=-\frac{2\pi \nu R}{\delta }\frac{q}{A},$$

where 0≤x≤L (cm) is the axial position along the vessel, μ (g cm^−1^ s^−1^) is the viscosity, ρ (g cm^−3^) is blood density, ν=μ/ρ (cm^2^ s^−1^) is the kinematic viscosity, R (cm) is the radius and t (s) is time. These equations rely on the assumption of a Stokes boundary layer


(2.6) $$\begin{array}{lcr} & u_{x}(r,x,t)=\left\{ \begin{array}{lr} {\bar{u}}_{x}, & r<R-\delta ,\\ {\bar{u}}_{x}\frac{\left(R-r\right)}{\delta }, & R-\delta <r\leq R. \end{array}\right. & \end{array}$$

Here, $\delta =\sqrt{\nu T/2\pi }$ (cm) is the boundary layer thickness, T (s) is the duration of the cardiac cycle, $u_{x}$ is the axial velocity and ${\bar{u}}_{x}$ is the axial velocity outside the boundary layer.

To close our system of equations, we define a pressure–area relationship of the form


(2.7) $$p(x,t)-p_{0}=\frac{4}{3}\frac{Eh}{r_{0}}\left(1-\sqrt{\frac{A_{0}}{A}}\right),$$

(2.8) $$\frac{Eh}{r_{0}}=k_{1}\exp(-k_{2}r_{0})+k_{3},$$

where E (g cm^−1^ s^−2^) is Young's modulus, h (cm) is the vessel wall thickness, $p_{0}$ (g cm^−1^ s^−2^) is a reference (e.g. unstressed state) pressure, $r_{0}$ (cm) is the unstressed inlet radius extracted from the MRA image segmentation at mid-diastole and $A_{0}$ (cm^2^) is the unstressed, cross-sectional area when $p(x,t)=p_{0}$ . The area $A_{0}=\pi r_{0}^{2}$ is determined from the vessel network as described in §2.2.

The system (2.4)–(2.5) is hyperbolic with Riemann invariants propagating in opposite directions. Therefore, boundary conditions are required at the inlet and outlet of each vessel. At the ascending aorta, corresponding to the root of the labelled tree, an inlet flow waveform is imposed using the patient-specific flow waveform extracted from the 4D-MRI data. At junctions, we impose mass conservation and pressure continuity


(2.9) $$q_{p}=q_{d1}+q_{d2},\;\;\;p_{p}=p_{d1}=p_{d2}.$$

The subscripts p and di,i=1,2 refer to the parent and daughter vessels, respectively. Outlet boundary conditions are imposed by coupling the terminals of the large vessels to an asymmetric structured tree model (described below) representing the small vessels [38]. The model equations are non-dimensionalized and solved using the explicit two-step Lax–Wendroff method [18,33,35,36,38].

### 2.3.2. Small vessels

In the small vessels, viscous forces dominate; therefore, we neglect the nonlinear inertial terms. As a result, equations (2.4) and (2.5) are linearized and reduced assuming periodicity of solutions, given by


(2.10) $$i\omega Q+\frac{A_{0}\left(1-F_{J}\right)}{\rho }\frac{\partial x}{\partial P}=0,\;\;\;F_{J}=\frac{2J_{1}(\omega_{0})}{\omega_{0}J_{0}(\omega_{0})},$$

(2.11) $$i\omega CP+\frac{\partial x}{\partial Q}=0,\;\;\;C=\frac{\partial A}{\partial p}\approx \frac{3}{2}\frac{r_{0}}{Eh},$$

where $J_{0}$ and $J_{1}$ are the zeroth- and first-order Bessel functions, $\omega_{0}^{2}=i^{3}r_{0}\omega /\nu$ is the Womersely number and C denotes the vessel compliance. The term $Eh/r_{0}$ has the same form as (2.8) but with small vessel stiffness parameters, $ks_{1},ks_{2}\text{ and }ks_{3}$ . Analytical solutions for equations (2.10) and (2.11) are


$$\begin{array}{rl} Q(x,\omega ) & =a\;\cos\left(\frac{\omega x}{c}\right)+b\;\sin\left(\frac{\omega x}{c}\right),\\ P(x,\omega ) & =\frac{i}{g_{\omega }}\left(-a\;\sin\left(\frac{\omega x}{c}\right)+b\;\cos\left(\frac{\omega x}{c}\right)\right), \end{array}$$

where a,b are integration constants and $g_{\omega }=\sqrt{CA_{0}(1-F_{J})/\rho }$ [18,33,36,38]. From this equation, it is possible to determine the impedance at the beginning of each vessel as a function of the impedance at the end of the vessel,


(2.12) $$\begin{array}{lcr} & Z(0,\omega )=\frac{ig^{-1}\sin\left(\frac{\omega L}{c}\right)+Z(L,\omega )\;\cos\left(\frac{\omega L}{c}\right)}{\;\cos\left(\frac{\omega L}{c}\right)+igZ(L,\omega )\;\sin\left(\frac{\omega L}{c}\right)}, & \end{array}$$

where $c=\sqrt{A_{0}(1-F_{J})/\rho C}$ is wave propagation velocity. Similar to large vessels, junction conditions impose pressure continuity and mass conservation.

The terminal impedance of each structured tree is assumed to be zero for all frequencies, and impedance is calculated through bifurcations recursively to compute the impedance at the root of each structured tree [38].

#### 2.3.3. Wave intensity analysis

Wave intensity is approximated using the pressure and flow predictions from the fluids model. The propagated pressure and flow waves are decomposed using wave intensity analysis (WIA), a tool that quantifies the incident and reflected components of these waves [18,39,40]. Components are extracted from the pressure and average velocity using


(2.13) $$\Gamma_{\pm }(t)=\Gamma_{0}+\int_{0}^{T}d\Gamma_{\pm },\Gamma =p,\bar{u},$$

(2.14) $$\begin{array}{lcr} & dp_{\pm }=\frac{1}{2}(dp\pm \rho c\;d\bar{u}),d{\bar{u}}_{\pm }=\frac{1}{2}\left(d\bar{u}\pm \frac{dp}{\rho c}\right), & \end{array}$$

where c (cm s^−1^) is the pulse wave velocity, dp the change in pressure and $d\bar{u}$ the change in average velocity across a wave [39]. The time-normalized wave intensity is given by


(2.15) $$\begin{array}{lcr} & WI_{\pm }=\left(\frac{\text{d}p_{\pm }}{\text{d}t}\right)\left(\frac{\text{d}{\bar{u}}_{\pm }}{\text{d}t}\right), & \end{array}$$

where the positive subscript refers to the incident wave and the negative to the reflective wave. Both incident and reflected waves can be classified as compressive or expansive. Compressive waves occur when $WI_{\pm },\text{d}p_{\pm }>0$ , while expansive waves occur when $WI_{\pm },\text{d}p_{\pm }<0$ . A wave-reflection coefficient is calculated by defining the ratio of amplitudes of reflected compressive pressure waves to incident compressive pressure waves


(2.16) $$\begin{array}{lcr} & I_{R}=\frac{\Delta p_{-}}{\Delta p_{+}}. & \end{array}$$

The incident (forward) wave begins at the network inlet. Forward compression waves (FCW) increase pressure and flow velocity as blood travels downstream to the outlet. Forward expansion waves (FEW) decrease pressure and flow velocity. Reflective (backward) waves begin at the outlet of the vessel. Backward compression waves (BCW) increase pressure and decrease flow velocity as blood is reflected upstream of the vessel while backward expansion waves (BEW) decrease pressure and accelerate flow [18,36].

#### 2.3.4. Wall shear stress

The stress, denoted $\tau_{w}$ (g cm^−1^ s^−2^), the fluid exerts on the vessel wall, called the wall shear stress (WSS), is defined using the Stokes boundary layer from equation (2.6)


$$\begin{array}{rl} \tau_{w} & =-\mu \frac{\partial u_{x}}{\partial r}\\ & =\left\{ \begin{array}{lr} 0, & r<R-\delta ,\\ \frac{\mu \bar{u}}{\delta }, & R-\delta <r\leq R, \end{array}\right. \end{array}$$

where μ is kinematic viscosity, δ the thickness of the boundary layer and $\bar{u}=q/A$ the average velocity [41].

### 2.4. Model summary

The one-dimensional CFD model, described in §2.3, predicts pressure, area and flow in all 17 large vessels within the network shown in figure 5. Solutions of the model rely on parameters that specify the fluid and vessel properties. The latter includes geometric parameters that determine the vessel lengths, radii and connectivity, material parameters that determine vessel stiffness and boundary condition parameters that determine inflow at the root of the network and outflow conditions at the terminal vessels. The model consists of both large vessels modelled explicitly and small vessels described by the structured tree framework.

#### 2.4.1. Fluid dynamics

Parameters required to specify the fluid dynamics include blood density ρ (cm^3^), dynamic μ (g cm^−1^ s^−1^) and kinematic ν=μ/ρ (cm^2^ s^−1^) viscosities and the boundary layer thickness $\delta =\sqrt{\nu T/2\pi }$ (cm).


$$\theta_{\text{fluid}}=\{\rho ,\mu ,\nu ,\delta \}.$$

Haematocrit and viscosity are assumed to be the same for all patients, and therefore, we keep these fixed for all simulations. Parameters ρ,μ and ν are used in the large and small vessels. Values for these parameters (table 3) are obtained from literature [38].

**Table 3.** Nominal parameter values for each patient group. D refers to the DORV group and H to the HLHS group. Parameters selected for inference are bolded. $r_{min}$ varies among the terminal vessels, so it is listed with a range.

parameter	description	unit	value (D)	value (H)
ρ	density	g cm^−^ ${,}^{3}$	1.06	1.06
μ	constant viscosity	g cm^−1^ s^−1^	0.032	0.032
ν	kinematic viscosity	cm ${,}^{2}$ s^−1^	0.030	0.030
δ	boundary layer thickness	cm	$\sqrt{\nu T/2\pi }$	$\sqrt{\nu T/2\pi }$
T	cardiac cycle length	s	0.710±0.090	0.770±0.190
$k_{1}$	large vessel stiffness	g cm^−1^ s^−^ ${,}^{2}$	$2.0\times 10^{7}$	$2.0\times 10^{7}$
$k_{2}$	large vessel stiffness	cm ${,}^{-1}$	−25.0	−35.0
$k_{3,g}$	large vessel stiffness	g cm^−1^ s^−^ ${,}^{2}$	$5.6\pm 1.1\times 10^{5}$	$7.3\pm 2.1\times 10^{5}$
$ks_{1}$	small vessel stiffness	g cm^−1^ s^−^ ${,}^{2}$	$2.0\times 10^{7}$	$2.0\times 10^{7}$
$ks_{2}$	small vessel stiffness	cm ${,}^{-1}$	−35.0	−30.0
$ks_{3,g}$	small vessel stiffness	g cm^−1^ s^−^ ${,}^{2}$	$3.8\pm 2.2\times 10^{5}$	$5.4\pm 2.1\times 10^{5}$
α	ST asymmetry constant	non-dim.	0.900	0.90−
β	ST asymmetry constant	non-dim.	0.600	0.600
lrr	length to radius ratio	non-dim	50.0	50.0
$r_{min}$	minimum radius	cm	0.001 - 0.010	0.001 - 0.010

#### 2.4.2. Geometry parameters

For each patient, geometric parameters are determined by segmenting the MRA images as described in §2.2. The network includes 17 vessels, so there are 3×17 parameters corresponding to vessel lengths ( L ), inlet radii $r_{\text{in}}$ , and outlet $r_{\text{out}}$ radii


$$\theta_{\text{geometry}}=\{r_{\text{in},i},r_{\text{out},i},L_{i}\},\;\;\;i=1,..17.$$

These are the unstressed vessel dimensions. Values for these parameters (table 2) are extracted from the imaging data and are kept constant for each patient.

#### 2.4.3. Material parameters

The model assumes that vessels have increasing stiffness with decreasing radii. This trend is modelled by equation (2.8), which contains three stiffness parameters: $k_{1}$ (g cm^−1^ s^−2^), $k_{2}$ (cm^−1^) and $k_{3}$ (g cm^−1^ s^−2^). Vessels are grouped by similarity, and we keep these parameters fixed within each group. Referring to figure 5, vessels 1, 2, 3, 4 and 7 are in group 1. Vessels 10, 11, 14 and 15 are in group 2, vessels 5, 12 and 13 are in group 3, and vessels 6, 8 ,9, 16 and 17 are in group 4. In summary, we have 12 material parameters for the large vessels


$$\theta_{\text{material}}=\{k_{1,g},k_{2,g},k_{3,g}\},$$

where subscript g=1,...,4 enumerates the vessel groups. In our previous study [18], these parameter values were determined using hand-tuning for a single DORV and HLHS patient pair. For this study, we use parameter values from [18] for $k_{1,g},k_{2,g}$ , while nominal values for $k_{3,g}$ are obtained by equations (2.7) and (2.8). Nominal parameter values are listed in table 3.

#### 2.4.4. Boundary condition parameters

The last set of parameters corresponds to either the inflow or the structured tree boundary conditions. For each patient, at the inlet of the network, an inflow waveform is extracted from the 4D-MRI image. For the outflow boundary conditions, seven structured tree parameters are needed for each terminal vessel (vessels 7, 8, 9, 12, 13, 14, 15, 16 and 17). Parameters include radius scaling factors α and β , which govern the asymmetry of the structured tree, lrr , which specifies the length-to-radius ratio and $r_{\text{min}}$ , the minimum radius used to determine the depth of the structured tree. In addition, the small vessels also have three stiffness parameters, $ks_{1},ks_{2}$ and $ks_{3}$ . Small vessel stiffness parameters are vessel specific: vessel 7, the descending aorta, is in group 1, vessels 10, 14 and 15 are in group 2, vessels 12 and 13 are in group 3, and vessels 8, 16 and 17 are in group 4. This gives a total of 16 structured tree parameters


$$\theta_{\text{boundary}}=\{ks_{1,g},ks_{2,g},ks_{3,g},\alpha ,\beta ,lrr,r_{\text{min}}\}.$$

Nominal values for $\alpha ,\beta ,lrr,r_{\text{min}},ks_{1,g}$ and $ks_{2,g}$ are taken from [18], and $ks_{3,g}$ is calculated in the same way as $k_{3,g}$ . For all terminal vessels except the descending aorta, the parameter $r_{\text{min}}$ is set to 0.001 cm. This value is consistent with the average radius of the arterioles. The parameter $r_{\text{min}}$ for the descending aorta is set to 0.01 cm, since this vessel is terminated at a larger radius.

#### 2.4.5. Quantity of interest

We estimate identifiable unknown parameters to quantify differences between the two patient groups. Our quantity of interest measures the discrepancy between model predictions and available data. Figure 4 marks locations for flow waveform measurements extracted from the ascending aorta, descending aorta, and the innominate, left common carotid and left subclavian arteries. Flow measured in the ascending aorta is used as the inflow boundary condition, and the remaining four flow waveforms are used to calibrate the model. We also have systolic and diastolic pressure measurements from the brachial artery. These are not measured simultaneously with the flows but are obtained in the supine position. Using this data, we construct residual vectors for both the flow and pressure. For each flow $q_{i}$ we compute


(2.17) $$\begin{array}{lcr} & r_{q_{i}}(t_{j})=\left[\frac{q_{i}^{m}(t_{j})-q_{i}^{d}(t_{j})}{q_{i,\max}^{d}}\right],i=1,\ldots 4,j=1,\ldots T_{p}, & \end{array}$$

where $r_{q_{i}}(t_{j})$ denotes the residuals between the flow data ( $q_{i}^{d}(t_{j})$ (ml s^−1^)) and the associated model predictions ( $q_{i}^{m}(t_{j})$ (ml s^−1^)) at each of the four locations ( i=1,…4 ) at time $t_{j}$ ( $j=1,\ldots T_{p}$ ), denoting the jth time step within the cardiac cycle. $T_{p}$ refers to the number of time point measurements (19–28) per cardiac cycle. This number differs between patients. The combined flow residual is defined as


(2.18) $$r_{q}=[r_{q_{1}}\;r_{q_{2}}\;r_{q_{3}}\;r_{q_{4}}],$$

where $r_{q_{i}}$ is the residual vector for flow i defined in (2.17). It should be noted that $r_{q}$ has dimensions of $4\times T_{p}$ .

Pressure measurements are available from one location at peak systole and diastole; therefore, the pressure residual is defined as


(2.19) $$r_{p}=[r_{p_{1}},r_{p_{2}}]=\left[\left(\frac{p_{\text{sys}}^{m}-p_{\text{sys}}^{d}}{p_{\text{sys}}^{d}}\right),\left(\frac{p_{\text{dia}}^{m}-p_{\text{dia}}^{d}}{p_{\text{dia}}^{d}}\right)\right],$$

where $p_{i}^{d}$ and $p_{i}^{m}$ , i=sys,dia (mmHg) denote the data and model predictions, respectively. As we do not have the exact location of the cuff measurement, we match model predictions to data at the midpoint of the brachial artery. We perform sensitivity and identifiability analyses to determine a subset of influential and identifiable parameters. The initial set of parameters to be explored includes those not determined from the patient data, i.e. the material and structured tree parameters,


(2.20) $$\theta_{\text{SA}}=\{k_{1,g},k_{2,g},k_{3,g},ks_{1,g},ks_{2,g},ks_{3,g},\alpha ,\beta ,lrr\}.$$

### 2.5. Sensitivity and identifiability analysis

Local sensitivity analysis provides insight into the influence of nominal parameters on the model's predictions. However, sensitivities can be inaccurate if the model is highly nonlinear and optimal parameters vary significantly from their nominal values. Global sensitivities provide additional information in the form of parameter influences over the entire parameter space. Local and global sensitivity analyses are performed on the flow residual vector $r_{q}$ defined in (2.18) with respect to parameters $\theta_{\text{SA}}$ defined in (2.20).

#### 2.5.1. Local sensitivity analysis

Using a derivative-based approach, we compute local sensitivities of the quantity of interest (residual vector) with respect to each parameter. Sensitivities are evaluated by varying one parameter and fixing all others at their nominal values [27]. We compute local sensitivities to log-scaled parameters $\left({\tilde{\theta }}_{\text{SA}}=\log\theta_{\text{SA}}\right)$ , ensuring both positivity and that parameter values are on the same scale. With these assumptions, the local sensitivity of $r_{q}$ , with respect to the nth component of the parameter vector, is given by


(2.21) $$\begin{array}{rl} {\tilde{S}}_{n} & =\frac{\partial r_{q}}{\partial {\tilde{\theta }}_{\text{SA}}^{n}}\\ & =\left[\frac{\partial q_{1}}{\partial \theta_{\text{SA}}^{n}}\frac{{\tilde{\theta }}^{n}}{q_{1,\max}^{d}},\ldots ,\frac{\partial q_{4}}{\partial \theta_{\text{SA}}^{n}}\frac{{\tilde{\theta }}^{n}}{q_{4,\max}^{d}}\right],n=1\ldots B, \end{array}$$

where B denotes the total number of parameters. Sensitivities are estimated using centred finite differences


(2.22) $$\frac{\partial r_{q}}{\partial \theta_{\text{SA}}^{n}}\approx \frac{r_{q}\left({\tilde{\theta }}_{SA}^{n}+e_{n}\psi \right)-r_{q}\left({\tilde{\theta }}_{SA}^{n}-e_{n}\psi \right)}{2\psi },$$

where ${\tilde{\theta }}_{\text{SA}}^{n}$ is the parameter of interest, ψ is the step size and $e_{n}$ is a unit basis vector in the nth direction [27,42]. Parameters are ranked from most to least influential by calculating the 2-norm of each sensitivity [27,31],


(2.23) $${\bar{S}}_{n}=||{\tilde{S}}_{n}|{|}_{2}.$$

#### 2.5.2. Global sensitivities

Morris screening [27,43,44] is used to compute global sensitivities. This method predicts elementary effects defined as


(2.24) $$d_{n}(\theta_{\text{SA}}^{n})=\frac{r_{q}(\theta_{\text{SA}}^{n}+e_{n}\Delta )-r_{q}(\theta_{\text{SA}}^{n})}{\Delta },$$

where the number of samples is set to K=100 and the number of levels of parameter space is set to M=60 , resulting in a step size of $\Delta =\frac{M}{2(M-1)}\approx 0.508$ . Elementary effects are determined by sampling K values from a uniform distribution for a particular parameter $\theta_{\text{SA}}^{n}$ . The elementary effects are ranked by computing the 2-norm of each effect


$${\tilde{d}}_{n}^{j}(\theta )=||d_{n}^{j}(\theta )|{|}_{2},$$

where j denotes the time point. Using the algorithm by Wentworth *et al*. [44], results are integrated to determine the mean and variance for the elementary effects. These are defined as


(2.25) $$\mu_{n}^{*}=\frac{1}{K}\sum_{j=1}^{K}|{\tilde{d}}_{n}^{j}|,$$

(2.26) $$\sigma_{n}^{2}=\frac{1}{K-1}\sum_{j=1}^{K}{\left({\tilde{d}}_{n}^{j}-\mu_{n}^{*}\right)}^{2},$$

where $\mu^{*}$ is the sensitivity of the quantity of interest and $\sigma^{2}$ is the variability in sensitivities due to parameter interactions and model nonlinearities. Parameters are ranked by computing $\sqrt{\mu^{*2}+\sigma^{2}}$ to account for the magnitude and variability of each elementary effect. To stay within a physiological range of parameter values, large and small vessel stiffness parameters are perturbed ±10% , and α,β,lrr are perturbed ±5% .

#### 2.5.3. Covariance analysis

Pairwise correlations between parameters can be determined using covariance analysis, constructing a covariance matrix


(2.27) $$\begin{array}{lcr} & v_{nj}=\frac{V_{nj}}{\sqrt{V_{nn}V_{jj}}}, & \end{array}$$

where $V=s^{2}[{\tilde{S}}_{n}^{T}{\tilde{S}}_{n}{]}^{-1}$ and $s^{2}$ is a constant observation variance. Studies using this method have defined correlated parameters for which $v_{nj}>0.8$ to 0.95 [27,45–47]. We assume that parameter pairs are correlated if $v_{nj}>0.9$ . The information gained from the sensitivity and covariance analyses allows us to determine a parameter set for inference, denoted $\theta_{\text{inf}}$ , which, as we will show in §3.2, is given by


(2.28) $$\theta_{\text{inf}}=\{\alpha ,k_{3,1:4},ks_{2},ks_{3,1:4}\}.$$

### 2.6. Parameter inference

The parameter subset $\theta_{\text{inf}}$ defined in equation (2.28) is inferred by minimizing the RSS,


(2.29) $$RSS=\sum_{i=1}^{4}\left(\sum_{j=1}^{T_{p}}r_{q_{i}}(t_{j}{)}^{2}\right)+\sum_{k\in \{\text{dia},\text{sys}\}}r_{p_{k}}^{2},$$

where $r_{q_{i}}(t_{j})$ and $r_{p_{k}}$ are defined in equations (2.17) and (2.19). To minimize equation (2.29), we use the sequential quadratic programming (SQP) method, a second-order gradient-based algorithm [27,48], which is implemented in Matlab’s fmincon function with the option ‘sqp’. Several previous studies have successfully used this algorithm on similar problems [35,40,49]. We use a tolerance of $1.0\times 10^{-8}$ [50]. Parameter bounds for each patient are set to ensure that model predictions remain within the physiological range. Exact nominal values and ranges for each patient are listed in the electronic supplementary material.

To complement the global sensitivity analysis, we use multistart inference to test whether the locally identifiable parameter subset remains identifiable. The optimizer is initialized to 12 sets of parameter values specified by sampling from a uniform distribution defined by varying the parameters ±30% of their nominal values. This range was informed by previous studies [18,31] and keeps parameters within a physiologically realistic range. We record the final value of the cost function and check for convergence across optimizations. Parameters with a coefficient of variation (CV, the s.d. divided by the mean) greater than 0.10 are removed from the subset. The multistart inference is repeated until all parameters in the subset converge and the CV for each parameter is below 0.10 . Parameters in the final subset, collected in the vector $\theta_{\text{inf}}$ , are estimated through one round of optimization with different initializations to avoid trapping in local minima.

## 3. Results

### 3.1. Image analysis

**Figure 6.**
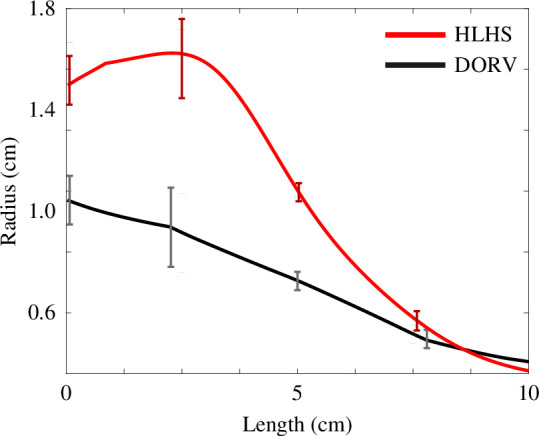
Aortic radii for a DORV and an HLHS patient pair. In the HLHS patient, the aortic radius increases along the ascending aorta and then decreases, while in the DORV patient, the aortic radius decreases along its length. Note that the two vessels have similar radii at the distal end. Error bars (one s.d. of radii measurements) are plotted at the inlet of the ascending aorta, aortic arch I, aortic arch II and descending aorta.

The DORV and HLHS patients have significantly different vessel radii. Figure 6 shows that remodelling of the reconstructed aorta in the HLHS group widens the ascending aorta and aortic arch as compared with the native aorta in the DORV group. For most HLHS patients, the aortic arch is wider than the ascending aorta. However, the two groups have approximately the same radii at the distal end of the descending aorta.

The geometric results in table 2 show that remodelling is heterogeneous. The variance of vessel dimensions (see electronic supplementary material for details) along the ascending aorta and aortic arch aorta is significantly smaller in the DORV group compared with the HLHS group, while the variance within each patient is similar (figure 6). Remodelling impacts the vessels in contact with the reconstructed tissue, namely the innominate artery and aortic arch. The aortic arch is widened, and the innominate artery is significantly shorter in HLHS patients. The same holds for the ascending aorta, which is also shorter in HLHS patients. The head and neck vessels and the descending aorta have similar dimensions and variance across groups, but as noted in §3.3, these vessels are stiffer in HLHS patients. For each patient type, vessel dimension ranges are listed in table 2, and exact vessel dimensions are reported in the electronic supplementary material.

### 3.2. Parameter inference

#### 3.2.1. Parameter identifiability

An identifiable parameter subset is constructed by combining the results of the local and global sensitivity analyses, covariance analysis and multistart inference.

**Figure 7.**
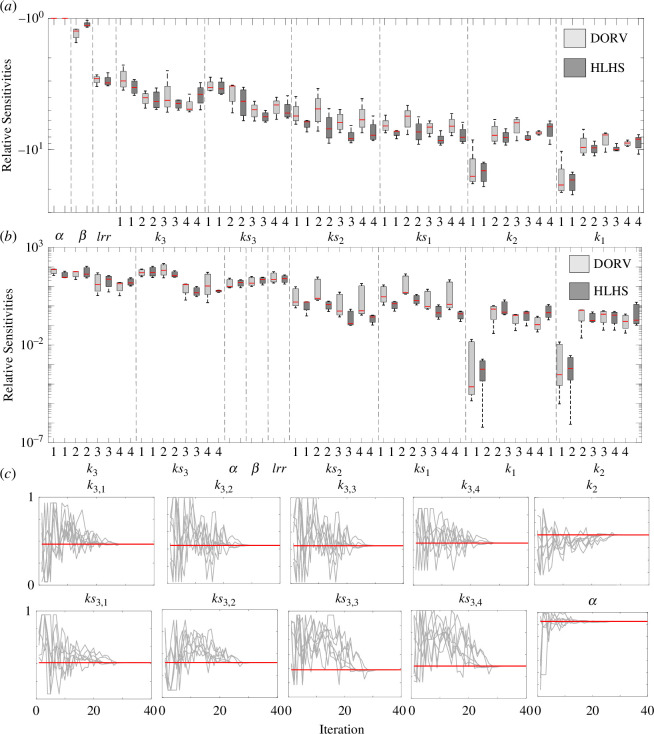
Sensitivity analysis and multistart inference. (*a*) Local sensitivities, (*b*) Morris screening and (*c*) multistart inference on the final identifiable parameter subset. Panels (*a*) and (*b*) are boxplots of the relative sensitivities for each patient group. Sensitivities are ranked from most to least influential. Panel (*c*) shows convergence of the final identifiable parameter subset. For each parameter, CV <0.1 . Note that parameters are scaled from 0 to 1 for easier comparison.

Local sensitivity analysis reveals that the most influential parameters are α , β and lrr . Other influential parameters include vessel stiffness parameters, $k_{3,g}$ , $ks_{3,g}$ and $ks_{2,g}$ (figure 7). Globally, $k_{3,g}$ and $ks_{3,g}$ are the most influential, followed by the structured tree parameters α,β,lrr . However, the relative sensitivities for these five parameters are similar, i.e. they are good candidates for the parameter subset. This result should be contrasted with parameters $k_{1,g}$ and $k_{2,g}$ , which are less influential.

Covariance analysis revealed a correlation between the structural tree parameters. Since α is the most influential parameter, we inferred α and fixed β and lrr . The large vessels stiffness parameters $k_{3,g}$ and $k_{1,g}$ are also correlated, and so are $k_{2,g}$ and $k_{1,g}$ . Informed by the sensitivity analyses, we chose to fix $k_{1,g}$ and $k_{2,g}$ and inferred only $k_{3,g}$ . For small vessels, $k_{2,g}$ is more influential, therefore, we fixed $ks_{1}$ and inferred $ks_{2,g}$ and $ks_{3,g}$ .

The initial parameter subset includes parameters $\theta_{\text{CA}}=\left\{\alpha ,k_{3,1:4},ks_{2,1:4},ks_{3,1:4}\right\}$ that are influential and locally uncorrelated. Multistart inference of $\theta_{\text{CA}}$ resulted in CV > 0.1 for $ks_{2,g}$ . Given that most structured trees are similar, we set $ks_{2,g}=ks_{2}$ . Repeated estimation including $ks_{2}$ as a global parameter gave an identifiable subset including $\theta_{\text{inf}}=\{\alpha ,k_{3,1:4},ks_{2},ks_{3,1:4}\}$ , i.e. all 12 samples estimated parameters with a CV < 0.1. Results of the multistart are shown in figure 7*c*.

#### 3.2.2. Model calibration

**Figure 8.**
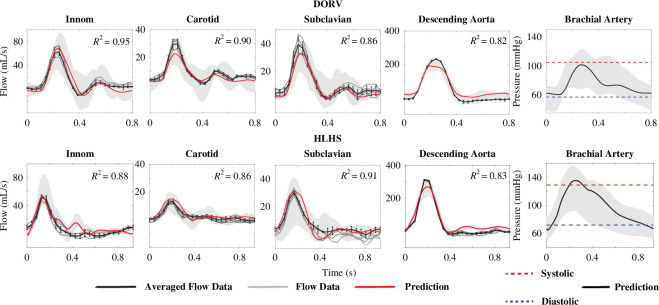
Model predictions versus patient data. From left to right, the first four panels in each row depict flow waveforms. The five grey lines denote the dynamics for each patient and the averaged dynamics are plotted with a black line. The black error bars represent one standard deviation of the averaged waveform. Model predictions are shown in red. The far-right panel in each row show the systolic (dashed red) and diastolic (dashed blue) cuff pressure measurements. Model predictions are depicted using solid black lines. Shading in the background of each panel denotes the results of simulations sampling the optimized parameters from a uniform distribution. $R^{2}$ values reporting the quality of model predictions are given in the top right of each panel.

Parameter inference results shown in figure 8 reveal that the model fits both the main and reflected features of the flow waveforms and the systolic and diastolic brachial pressures. Optimal model predictions are plotted with a solid red line and the averaged measured flow waveforms are with solid black lines. Error bars were obtained by averaging waveforms extracted at nearby points within the vessel, as described in §2.1. These bars correspond to one s.d. above and below the mean. The grey silhouette represents predictions generated by sampling parameters from a uniform distribution within the bounds used for parameter inference, demonstrating the ability of the optimization to generate model outputs that fit the data. For flow waveforms, $R^{2}$ values were always greater than 0.80, indicating the model captures at least 80% of variability in the data [51].

### 3.3. Model predictions

#### 3.3.1. Inferred parameters

**Figure 9.**
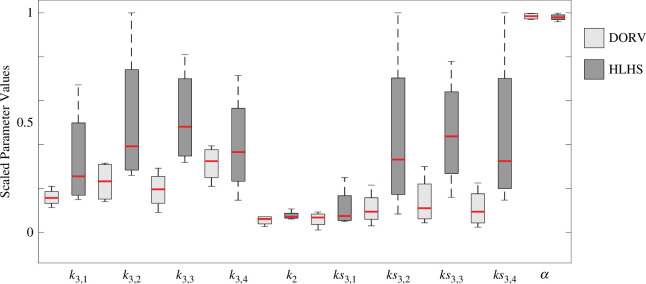
Box and Whisker comparison of inferred parameter values. Light grey boxes represent the DORV patients, while dark grey represents the HLHS group. Parameters are scaled from 0 to 1 for easier comparison.

Figure 9 compares the estimated parameters, scaled from 0 to 1 . These results show that the aortic stiffness ( $k_{3,1}$ ) is higher and more variable in HLHS patients, which appears to be consistent with our results for the vessel geometry. Even though vessels further from the reconstruction have similar geometry between patient groups, vessel stiffness ( $k_{3,2:4}$ ) is increased in HLHS, indicating remodelling affects the vasculature as a whole. Moreover, peripheral vessels are stiffer in HLHS patients, but remodelling has not affected the peripheral branching structure (estimated values for α are the same for all vessels). This result seems to agree with the finding that geometry is not altered in the peripheral vessels.

#### 3.3.2. Flow, pressure and area predictions

**Figure 10.**
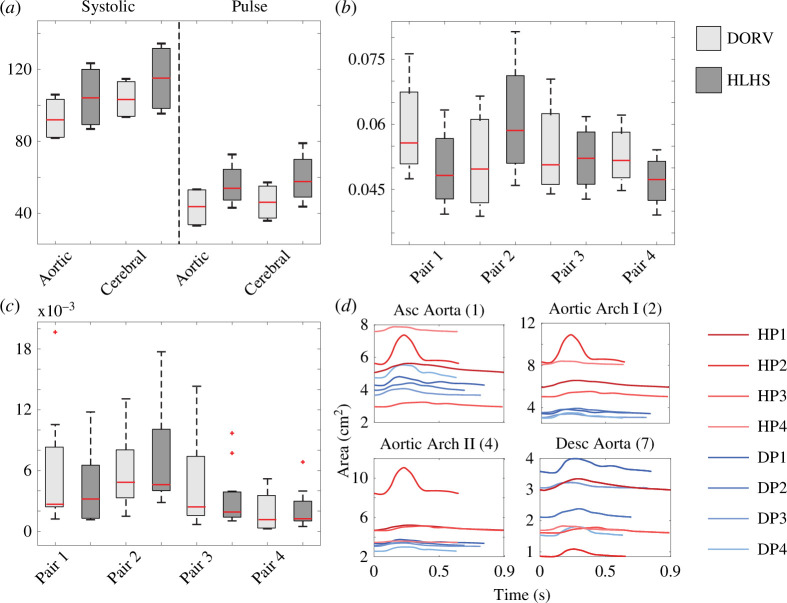
Model predictions. (*a*) The average systolic and pulse pressure in the aorta and cerebral vessels. Pairwise comparison of the average pulse flow in the aorta (*b*) and cerebral (*c*) vessels. Pulse flows are shown relative to total cardiac output. (*d*) Area deformation over a cardiac cycle, HLHS patients are shown in red and DORV in blue.

Figure 10*a* shows that average systolic and pulse pressures are higher in the aortic and cerebral arteries for HLHS patients. Figure 10*b,c* shows pairwise comparisons of flow predictions. Averaging of all patients within each group shows no differences, but pairwise comparisons of pulse flow (max flow − min flow) reveal different trends. In (*b*), the pulse flow is lower in HLHS patients or has no significant difference in aortic vessels, except in HLHS patient 2 (from patient pair 2). In (*c*), on average, the pulse flow is decreased in the HLHS cerebral vasculature. This can be seen better by comparing the values vessel-wise (see electronic supplementary material). Paired HLHS and DORV patients have similar cardiac outputs; therefore, this pairwise comparison provides greater insight. Larger pulse flows in cerebral vessels indicate greater perfusion to the brain, while similar pulse flows in the descending aorta suggest both groups are possibly at the same risk for FALD (see electronic supplementary material). Note that the reported pulse flows are relative to the total cardiac output. Figure 10*d* shows that HLHS patients’ aortic vessels deform less over a cardiac cycle compared with DORV.

#### 3.3.4. Wave intensity analysis

**Figure 11.**
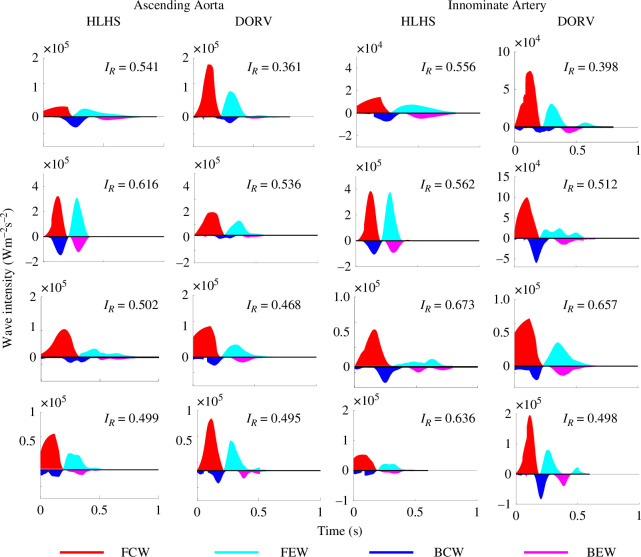
Reflected backward compression waves (BCW) and backward expansion waves (BEW). Incident forward compression waves (FCW) and forward expansion waves (FEW) in the ascending aorta and left/right carotid artery for each patient. The patterns in the other aortic and peripheral vessel segments are similar to the vessels shown here. Each panel also lists the patient's wave-reflection coefficient, $I_{R}$ .

The incident and reflected waves shown in figure 11 differ significantly between the two patient groups. Overall, the wave-reflection coefficient $I_{r}$ is higher in HLHS patients, suggesting reconstructed aortas induce more reflections. This might be in part due to larger differences in vessel size and stiffer vessels for patients in the HLHS group. Moreover, DORV patients have significantly higher forward compression and expansion waves, and HLHS patients have higher backward compression waves, probably as a result of the widened ascending aorta and aortic arch, increasing the wave reflections. The exception to this trend is pair number 2. The DORV patients do not differ significantly from the other patients in the group, but HLHS patient 2 has significantly higher forward and backward waves. This patient does not have significantly different geometry when compared with the other patients. However, they do have increased pulse flow in their aortic vessels. Our modelling approach can predict abnormalities in this patient by taking into account the complex interactions of these quantities.

#### 3.3.5. Wall shear stress

**Figure 12.**
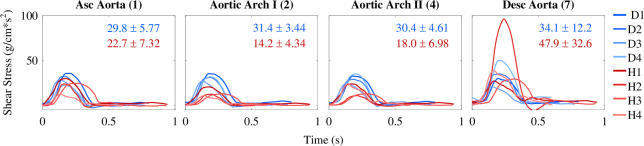
Wall shear stress in the four aortic vessels for each patient. Shades of blue represent DORV patients, and shades of red represent HLHS patients. The average ± 1 s.d. peak WSS for the DORV and HLHS groups is noted on the graphs. DORV patients' WSS decreases slightly away from the heart, while HLHS patients' WSS increases. For all patients, the WSS is significantly higher in the descending artery. HLHS patient 2 has considerably higher descending aorta WSS than the other patients.

We computed WSS in the centre of the aortic vessel segment for each patient (figure 12). DORV patients have higher WSS values that peak between 25 and 50 g cm^−1^ s^−2^ compared with HLHS patients, with WSS values peaking between 10 and 29 g cm^−1^ s^−2^. All patients have similar WSS values in the ascending aorta and aortic arch, but they are significantly higher in the DORV patients in these regions. Both groups have increased wall shear stress in the descending aorta, but the increase is higher in HLHS patients. This is probably a result of the stiffening of the descending aorta in this patient group. Again, HLHS patient 2 has a larger WSS in the descending aorta compared with the other patients.

## 4. Discussion

This study describes a framework for building patient-specific one-dimensional CFD models and applies these models to predict haemodynamics for DORV and HLHS single-ventricle patients. We analyse medical images to extract patient geometries and quantify differences in vessel dimensions; perform local and global sensitivity analyses, covariance analysis and multistart inference to determine an influential and identifiable subset of parameters. We infer this subset, $\theta_{\text{inf}}=\left\{k_{3,g},ks_{2},ks_{3,g},\alpha \right\}$ . Results show that HLHS patients have wider ascending aortas, increased vascular stiffness throughout the network, increased pressures in the aorta and cerebral vasculature and increased wave reflections. Additionally, model predictions reveal that HLHS patient 2 has quantitatively different pressure and flow patterns compared with the other HLHS patients.

### 4.1. Image analysis

Patient geometry derived from medical imaging data provide known parameters that are integrated into the model. It has a substantial influence on the model predictions. Colebank *et al*. [35] and Bartolo *et al*. [33] detail the importance of accurate vascular geometry and its influence on model predictions. HLHS patients have greater ascending aorta radii. Two HLHS patients have significant remodelling, with ascending aortas increasing along the aortic arch rather than decreasing. However, all patients have similarly sized descending aortas. This abnormal geometry inherent to the HLHS cohort probably contributes to abnormal flow patterns and disturbances, promoting continued vascular and ventricular remodelling over time [52–54]. Hayama *et al*. [54] noted that aortic roots in HLHS patients continue to dilate over time, contributing to both ventricular and downstream remodelling and eventual Fontan failure. Therefore, monitoring the change in aortic geometry over time is essential for single-ventricle patients with reconstructed aortas.

### 4.2. Parameter inference

Local sensitivity analysis reveals that the structured tree parameters α and β have the greatest influence on the quantity of interest. Stiffness parameters $k_{3,g}$ and $ks_{3,g}$ also influence the predictions significantly. This result agrees with findings by Paun *et al*. [55] who used a similar one-dimensional CFD model. Their study only considered local sensitivities, and parameters were not vessel specific. They concluded that α was the most influential, followed by β and $k_{3}$ . Our findings also agree with work from Clipp and Steele [56], which emphasized the importance of tuning the structured tree boundary condition parameters to fit the model to data.

Morris screening results are consistent with local sensitivity results in the sense that the five most influential parameters are the same in each case. Figure 7 shows that while $k_{3,g}$ and $ks_{3,g}$ are slightly more influential than the structured tree parameters, there are no significant differences in the values of the relative sensitivities. Our findings are also consistent with experimental studies investigating the effects of aortic stiffness and resistance on haemodynamics. In the aortic vasculature, it has been found that increased aortic stiffness can decrease stroke volume and increase pressure [57,58]. Through an *in vitro* investigation focused on the impact of aortic stiffness on blood flow, Gülan *et al*. [58] found that increasing aortic stiffness greatly influences velocity patterns and blood volume through the aorta.

Covariance analysis revealed a correlation between the structured tree parameters, as well as between $k_{1,g}$ and the stiffness parameters $k_{2,g}$ and $k_{3,g}$ . We inferred α and fixed β and lrr . The parameter α is the most influential, and it is not correlated with any of the stiffness parameters. We fixed $k_{1}$ and $k_{2}$ for all vessels to reduce the number of inferred parameters. Covariance analysis is performed using the local sensitivity matrix; therefore, there is no guarantee that parameters remain correlated after being estimated. Multistart inference results showed that $ks_{2,g}$ had a CV greater than 0.1, and estimated parameters varied significantly. Due to the influence that $ks_{2}$ has on the model, we inferred that parameter but used a global rather than vessel-specific value. These findings are consistent with studies from Colebank & Chesler [59] and Paun *et al*. [55] in which they performed related analyses on similar models and chose to infer large artery stiffness, small vessel stiffness and at least one structured tree parameter.

Figure 9 compares the inferred parameter values between the two patient groups. Overall, DORV patients have lower large vessel stiffness in the aorta and peripheral vessels. The stiffness varies less between vessel types, and haemodynamic predictions are more uniform. Estimated values for the α and $ks_{2}$ parameters did not differ significantly between groups. However, small and large vessel stiffness, $ks_{3,g}$ and $k_{3}$ , respectively, were substantially higher in the HLHS group, with DORV patients having lower downstream resistance vascular stiffness than HLHS patients. This finding is consistent with those from Cardis *et al*. [60] and Schafer *et al*. [61], who found that Fontan patients with HLHS had higher vascular stiffness compared with other Fontan patient types. The increased stiffness might occur in part due to the properties of the non-native tissue used to surgically reconstruct the aorta [60]. For most HLHS patients, reconstruction is performed with a homograft material that comprises at least 50% of the reconstructed vessel [60]. This homograft material differs significantly from the native aortic tissue, and remodelling over time generates tissue that is significantly stiffer than the native aorta [62,63]. On a related note, clinicians have discussed the utility of aortic stiffness in helping to determine when medical intervention is needed. The retrospective study by Hayama *et al*. [54] found that increased aortic stiffness in Fontan patients is correlated with exercise intolerance, vascular and ventricular remodelling, and heart failure. They postulated that surgical intervention and vasodilation/hypertension medication may help offset vascular remodelling [54].

### 4.3. Model predictions

#### 4.3.1. Haemodynamic predictions

Figure 9 demonstrates that our parameter inference method generates model predictions that fit the data reasonably well. We found that the HLHS group has increased average systolic and pulse pressures with the interquartile range (IQR) also being higher in aortic and cerebral vasculature. Given the heterogeneous geometry present in the HLHS group and the small sample size, the latter is only evident with comparisons between patient pairs. Since HLHS requires surgical interventions over multiple years, we found that using matched DORV patients provided a clear and systematic way to understand the effects of surgical aortic reconstruction and subsequent remodelling on model-predicted haemodynamics. All HLHS patients are hypertensive [64] according to model predictions, despite three out of four HLHS patients receiving medication to reduce blood pressure. In particular, cerebral blood pressure was high. Decreased pulse flows are consistent with research that describes inadequate perfusion and oxygen transport to the brain in HLHS patients with reconstructed aortas [10,65,66]. Brain perfusion appears to be an important clinical endpoint, since it has recently been shown that HLHS patients have abnormal cerebral microstructure and delayed intra-uterine brain growth [67,68]. To vessel area deformation over a cardiac cycle, the aortic vessels in the HLHS patients (except for HLHS patient 2) deform less than the same vessels in the DORV patients (see electronic supplementary material). Vessels with small deformations over a cardiac cycle tend to have increased stiffness and tend to appear in patients with larger aortic radii and abnormal wall properties [69].

#### 4.3.2. Wave intensity analysis

Figure 11 shows that the DORV patients have larger forward waves than their reflective, but HLHS patients have smaller forward waves and increased reflective waves. In HLHS patients, the ascending aorta has smaller forward waves and larger backward waves compared with the DORV group. Wave-reflection coefficients $I_{R}$ (figure 11) confirm these differences. For each matched patient pair, the HLHS patient has a larger $I_{R}$ . The $I_{R}$ values we found for DORV patients agree with those reported in the literature [70]. The results of our WIA for the ascending aorta of the HLHS group are consistent with a similar analysis by Schafer *et al*. [71], who noted increased ratios of backward to forward waves in HLHS patients with reconstructed aortas. Increased backward compression waves in the ascending aorta indicate a sudden deceleration of the forward blood flow wave, leading to an increase in afterload and a decrease in ventricular performance [71]. In particular, HLHS patient 2 has a significantly higher $I_{R}$ in the aortic vessels compared with the other patients. These results signify abnormal blood flow within the aorta of HLHS patients, possibly indicating ventricular decline.

#### 4.3.3. Wall shear stress

Except for DORV patient 3, WSS peak values in the DORV group decreased in the descending aorta compared with the ascending aorta. This finding is consistent with studies using CFD models to compute WSS in healthy patients [72,73]. The HLHS patients has lower WSS values in the ascending aorta and aortic arch compared with the DORV patients. Reduced WSS can be indicative of hypertension and stiffening of the vessel wall. Traub & Berk [74] found that consistently low WSS values were correlated with upregulation of vasoconstrictive genes. This promoted smooth muscle cell growth, which led to a loss of vessel compliance. For the larger WSS values in the descending aorta, Voges *et al*. [75] found that the descending aorta, in HLHS patients with reconstructed aortas, dilates over time due to increased WSS. Notably, HLHS patient 2 has a large increase in WSS in the descending aorta. Of the HLHS group, this patient has the smallest inlet radius for the descending aorta. As the body begins to remodel, this WSS could decrease as the inlet radius widens. WSS is an important clinical marker that cannot be measured *in vivo*. However, it can be determined from CFD models, as demonstrated by Loke *et al*. [76]. They used CFD modelling and surgeon input to develop a Fontan conduit that minimized power loss and shear stress, thereby improving flow from the gut to the pulmonary circuit. Many studies have focused on WSS in the Fontan conduit and pulmonary arteries [77,78]. However, there is a lack of work devoted to studying WSS within the aorta and systemic arterial vasculature for single-ventricle patients. The results reported here give crucial insight into haemodynamics and information on the degree of remodelling.

### 4.4. Future work and limitations

This study describes the construction of patient-specific, one-dimensional CFD arterial network models that include the aorta and head/neck vessels for four DORV and four HLHS patients. A main contribution is the development of a parameter inference methodology that uses multiple datasets. To obtain reliable parameter inference results, we limited the number of vessels in the network. The small size of the network makes it challenging to predict cerebral and gut perfusion. A way to overcome this limitation is to add more vessels, e.g. to use the network defined in the study by Taylor-LaPole et al. [18]. Another limitation is that HLHS patients generally have abnormal aortic geometries due to surgical reconstruction. These geometries most likely cause energy losses as blood flows from the wider arch into the narrow descending aorta. Our model does not predict these energy losses. However, previous studies have included energy loss terms in one-dimensional arterial network models [36,79]. This approach could be adapted for this study, but more work is needed to calibrate the parameters required for these energy loss models. Calibration could be informed by the analysis of velocity patterns from 4D-MRI images or three-dimensional fluid–structure interaction models.

The Fontan circuit has been the subject of many studies using three-dimensional CFD models [23–25]. While these models are excellent for analysing complex velocity patterns, they are limited to the imaged region and are generally computationally intensive. In contrast, one-dimensional CFD models offer an efficient and accurate alternative. Several studies have shown that the flow and pressure waves predicted by one-dimensional models are comparable to those obtained with three-dimensional models [80–82], validating the use of one-dimensional models. Future research will explore optimization techniques that allow this model to be used in real-time clinical decision-making, a possibility made feasible by the efficiency of the one-dimensional model over three-dimensional modelling.

This study minimizes the Euclidean distance between measurements and model predictions inferring biophysical parameters of interest and ignoring the correlation structure of the measurement errors due to the temporal nature of the data. In future studies, we aim to capture the correlation by assuming a full covariance matrix for the errors by using Gaussian processes [83]. We will also incorporate model mismatch to account for discrepancies between data and model predictions (figure 8) caused by numerical errors or model assumptions by using the methodology presented in [83]. We did not include analyses of high-order interaction from multiple parameters being considered; this will be explored in future work. Also, our sensitivity analyses were performed with respect to $r_{q}$ as our data consisted mainly of flow waveforms. On one patient, we did perform these same analyses, including $r_{p}$ (data not shown), and this method was slightly less efficient and did not produce different results. Finally, many modelling studies devoted to the Fontan circulation focus only on venous haemodynamics, in part because venous congestion impacts blood returning to the heart from the liver and probably contributes to the progression of FALD. In the future, a two-sided vessel network model could be incorporated into this framework that includes a description of both the arterial and venous vasculature (e.g. [26]).

There are limitations related to the clinical data that were used in this study. The 4D-MRI images are averaged over several cardiac cycles. This averaging and noise in the scans probably contribute to a lack of volume conservation in the flow data created from these images. We performed parameter inference using one pressure reading from one vessel. In the future, it would be helpful for parameter estimation to have multiple pressure readings from multiple vessels, e.g. from the arms and the ankles. We do realize the sample size is small—limiting our ability to validate our model. It is our plan in the future to obtain access to more patient data and validate results in this way. Finally, five out of the nine patients received some form of hypertension medication. This is a factor that our model does not take into account.

## 5. Conclusions

This study defines a patient-specific one-dimensional CFD model and a parameter inference methodology to calibrate the model to 4D-MRI velocity data and sphygmomanometer pressure data. Results from the parameter inference give insight into physiological phenomena such as vascular stiffness and downstream resistance. The reconstructed aortas in the HLHS patients were wider than the native aortas of the DORV patients, and parameter inference revealed that HLHS patients had increased vascular stiffness and downstream resistance. Model predictions showed that vessels in HLHS patients do not distend over a cardiac cycle as much as those in DORV patients, indicative of hypertension. WIA predicted increased backward waves in the ascending aorta of HLHS patients, suggesting abnormal blood flow. Results show decreased WSS in HLHS patients indicative of hypertension and a precursor to remodelling. HLHS patient 2 in particular has the highest pressures, largest backward waves, and largest WSS of the HLHS patient group, indicating this patient may require additional clinical, possibly surgical, intervention. To our knowledge, this study is the first patient-specific one-dimensional CFD model of the Fontan systemic arterial vasculature that is calibrated using multiple datasets from multiple patients.

## Data Availability

All data has been included in the electronic supplementary material. Code for the fluids simulations, sensitivity analysis and parameter inference can be found at [84]. Supplementary material is available online [85].
