# Correction: ‘Parameter selection and optimization of a computational network model of blood flow in single-ventricle patients’ (2025), by Taylor-LaPole

**DOI:** 10.1098/rsif.2025.0649

**Published:** 2025-11-05

**Authors:** Alyssa M. LaPole, Mihaela Paun, Dan Lior, Justin Weigand, Charles Puelz, Mette S. Olufsen

**Affiliations:** ^1^ Department of Biomathematics, Rice University, Houston, TX 77005-1892, USA; ^2^ Department of Mathematics and Statistics, University of Glasgow, Glasgow G12 8QQ, UK; ^3^ Department of Pediatrics, Baylor College of Medicine, Houston, TX 77030-3411, USA; ^4^ Division of Cardiology, Department of Pediatrics, Baylor College of Medicine, Houston, TX 77030-3411, USA; ^5^ Department of Mathematics, North Carolina State University, Raleigh, NC, USA

*J. R. Soc. Interface*. 22, 20240663. (Published online 27 February 2025). (https://doi.org/10.1098/rsif.2024.0663)

We have identified one error in the manuscript and made the following correction:

(1) Correction of figure 8. The averaged data waveform (black) was plotted against the incorrect raw data waveforms (grey) in the HLHS subclavian panel (bottom centre panel). The figure has been updated accordingly.

**Figure 9.**
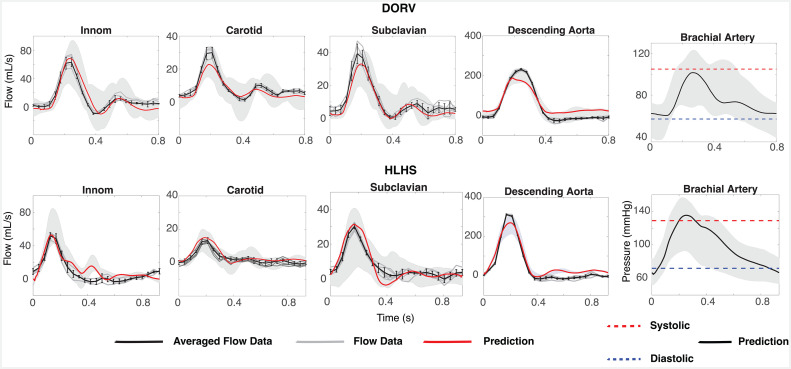
Model predictions versus patient data. From left to right, the first four panels in each row depict flow waveforms. The five grey lines denote the dynamics for each patient and the averaged dynamics are plotted with a black line. The black error bars represent one standard deviation of the averaged waveform. Model predictions are shown in red. The far-right panel in each row show the systolic (dashed red) and diastolic (dashed blue) cuff pressure measurements. Model predictions are depicted using solid black lines. Shading in the background of each panel denotes the results of simulations sampling the optimized parameters from a uniform distribution. $R^{2}$ values reporting the quality of model predictions are given in the top right of each panel.

